# The Next Generation of FACS – Not Just a Uniform Blob of Fluorescence

**DOI:** 10.1097/HS9.0000000000000709

**Published:** 2022-03-31

**Authors:** David G. Kent

**Affiliations:** York Biomedical Research Institute, Department of Biology, University of York, United Kingdom

Fluorescence-activated cell sorting (FACS) has been a fundamental technology across hematology and immunology labs for over 50 years. In his original article about the cell sorter published in 1965, Mack Fulwyler imagined “it may be possible to also measure simultaneously two (or more) characteristics of a cell and to make separation dependent on the ratio of such characteristics.”^1^ Fast forward to today, and we have dozens of FACS machines that can separate tens of thousands of cells per second based on combinatorial expression of >15 markers and some that even press beyond this. But scale is not the only thing that matters and a recent article by Schraivogel et al^2^ highlights a new FACS-based technology that launches us into the next galaxy of possibilities—spatial sorting.

The technology adaptation in question has been dubbed “image-enabled cell sorting” or ICS and in essence it takes (and processes) high-speed images alongside the normal cell sorting parameters of laser-activated fluorescent labels. It can collect images of a wide array of physical features and for some features, it enables sorting of cell populations based on these spatial parameters (eg, single or multiple/enlarged nucleoli, single or multiple nuclei, and different cell shapes). For those familiar with the ImageStream technology, this new system is the same concept (images and flow cytometry), but it is much faster and it can sort viable cells. A key component is undertaking the imaging using fluorescent radiofrequency-tagged emission (FIRE), a fast fluorescence imaging technique. Very briefly, it involves splitting a laser beam into multiple beams and combining them with light from another laser to reconstruct an image based on distinct interference patterns (see original *Nature Photonics* article for a complete explanation).^3^

One striking example of the technology’s power was highlighted in its application to sorting cells in the different phases of the cell cycle. Whereas current FACS machines can sort cells based on G_0_/G_1_; G_2_/mitosis; and -phase, ICS is able to separate additional stages based on a combination of morphology and fluorescence with relatively high accuracy: G2 interphase (96% purity), prometaphase (64% purity), metaphase (78% purity), anaphase (94% purity), and telophase (93% purity). Researchers currently using chemical methods to synchronize the cell cycle status of their population may be major beneficiaries of this technology that could greatly enrich cells for specific cell cycle stages without the need for perturbations that could also cause additional molecular and/or biochemical alterations.

A second incredible vignette was utilizing ICS for undertaking functional genomic screens. Using RelA nuclear translocation as a readout for NF-κB pathway activation—something that is not able to be assessed by nonimaging FACS—the authors undertook a CRISPR-based guide RNA screen of ~1000 known and suspected NF-κB regulators at ×100 coverage in <9 minutes. Next, they moved the technique to a genome-wide CRISPR screen involving 3 guide RNAs per gene and again with ×100 coverage and were able to screen the entire library in 9 hours of machine run time. Following the identification of new candidate regulators, they undertook validation assays using individual CRISPR knockouts and showed strong correlation between microscopy and ICS technologies. Researchers can currently undertake versions of image-based CRISPR screens with microscopy tools, but the scale and speed of image analysis is virtually impossible to imagine using conventional microscopes.

The speed of cell sorting, in combination with super-fast imaging, now enables screens that require an image-based readout (eg, nuclear translocation, asymmetric partitioning, etc.) to be undertaken. It will further permit a huge range of other functional assays of rare cell populations with distinct morphological or spatial characteristics. If the technology can continue to get faster and be optimized to run as quickly as a traditional nonimage based FACS machine, then this technology will be a real game-changer for the field and it is exciting to imagine all the future possibilities of what can be studied in cellular systems from a huge range of different fields.

## DISCLOSURES

The author has no conflicts of interest to disclose.

## References

[1] Fulwyler MJ. Electronic separation of biological cells by volume. Science. 1965;150:910–911. 5891056 10.1126/science.150.3698.910

[2] Schraivogel D Kuhn TM Rauscher B. High-speed fluorescence image-enabled cell sorting. Science. 2022;375:315–320. 35050652 10.1126/science.abj3013 PMC7613231

[3] Diebold E Buckley BW Gossett DR Jalali B. Digitally synthesized beat frequency multiplexing for sub-millisecond fluorescence microscopy. Nature Photon. 2013;7:806–810.

